# Metasurfaces enable sculpting light in three dimensions

**DOI:** 10.1038/s41377-026-02239-z

**Published:** 2026-03-03

**Authors:** Joohoon Kim, Junsuk Rho

**Affiliations:** 1 Department of Mechanical Engineering, Pohang University of Science and Technology (POSTECH), Pohang, 37673 Republic of Korea; 2 POSTECH Institute of Artificial Intelligence (PIAI), Pohang University of Science and Technology (POSTECH), Pohang, 37673 Republic of Korea; 3 Department of Chemical Engineering, Pohang University of Science and Technology (POSTECH), Pohang, 37673 Republic of Korea; 4 Department of Electrical Engineering, Pohang University of Science and Technology (POSTECH), Pohang, 37673 Republic of Korea; 5 POSCO-POSTECH-RIST Convergence Research Center for Flat Optics and Metaphotonics, Pohang, 37673 Republic of Korea

**Keywords:** Metamaterials, Photonic devices

## Abstract

A recent study demonstrates a metasurface platform for 3D vectorial holography that enables independent control of light intensity and polarization along the propagation axis. By utilizing longitudinally engineered meta-atoms, this approach achieves multi-dimensional optical encryption platform.

Holography has long been recognized as a key technology for virtual reality, data security, and optical information processing^1,2^. The advent of computer-generated holography (CGH) marked a paradigm shift, moving beyond traditional light-field recording on photosensitive materials to the proactive design of complex wavefronts^3,4^. This transition has enabled the optimization of wavefront with significantly higher degrees of freedom, primarily focusing on tailoring light intensity distributions in two-dimensional (2D) planes. Leveraging this increased flexibility, metasurface holograms (metaholograms) have successfully demonstrated advanced functionalities^5^, including polarization-multiplexed holography^6^, orbital angular momentum (OAM)-multiplexed holography^7,8^, propagation-dependent three-dimensional (3D) holography^9^, full-color RGB displays^10,11^.

Building upon these foundations, vectorial holography has emerged as a powerful tool by exploiting the polarization degree of freedom to enhance information capacity^12^. Unlike scalar holograms that only modulate phase or amplitude, vectorial holograms utilize spatially varying meta-atoms to locally control the polarization state of light, enabling the reconstruction of different holographic images depending on the polarization state of light, enabling the reconstruction of different holographic images depending on the incident or analyzed polarization. While these developments have significantly expanded the functionality of metasurfaces, achieving simultaneous and independent control of both intensity and polarization within a 3D volumetric space has remained a formidable challenge. The inherent complexity of phase-polarization coupling and longitudinal interference has hindered the full realization and practical implementation of such intricate 3D vectorial holograms.

Recently, a research team led by Prof. Ting Xu addressed this challenge in their study published in *Light: Science & Applications* by demonstrating longitudinally engineered metasurfaces that achieve independent control over both intensity and polarization in 3D space^13^. To achieve this, Tan et al. went beyond conventional phase modulation by exploiting the full potential of meta-atom design. As illustrated in Fig. 1a, the researchers meticulously optimized the geometric parameters and spatial arrangements of meta-atoms, allowing each unit cell to function as a localized Jones matrix element.

**Fig. 1:**
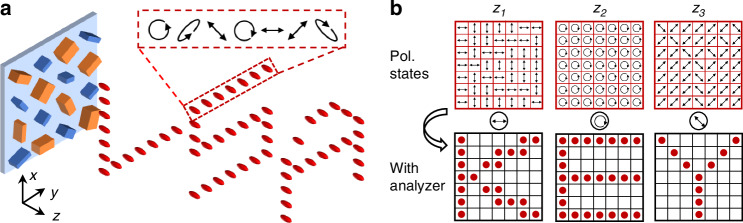
Conceptual illustration of 3D vectorial holography via longitudinally engineered metasurfaces. **a** Schematic showing the generation of 3D volumetric patterns with tailored intensity profiles and polarization states. **b** Principle of polarization-gated decoding. At specific axial planes (z_1_, z_2_, z_3_), the encoded information (e.g., characters “K”, “E”, “Y”) is revealed only when viewed through the matching polarization analyzer

The core principle involves the precise mapping of the required complex amplitude and polarization states along the propagation axis (*z*-axis). By decoupling the geometric phase from the propagation phase, the metasurface can independently dictate the evolution of light’s intensity profile and its polarization trajectory, often visualized as a path on the Poincare sphere, at every pixelated point within a 3D volume. This dual-layer modulation effectively “sculpts” the light field in 3D space, realizing a 3D vectorial hologram where information is no longer confined to a single plane but is intricately distributed across space.

The most compelling demonstration of this 3D control is the implementation of polarization-gated optical encryption (Fig. 1b). The proposed metasurface generates a light array where, in the absence of a polarizer, the intensity appears uniform across a specific plane, rendering any encoded information invisible to the naked eye. However, when the correct polarizer is applied, it selectively filters the pre-designed polarization states, allowing the hidden characters to emerge with high contrast. This mechanism establishes a significantly higher level of data security compared to traditional methods. To retrieve the encrypted information, an authorized user must possess the precise “keys”, the exact spatial coordinates (*z*-position) and the corresponding polarization parameters (slow axis and fast axis in Poincare sphere). By transforming both spatial position and polarization into indispensable security factors, Tan et al. have proposed a robust multi-dimensional security platform that goes far beyond simple image projection.

While the current demonstration utilizes a Bessel beam-based holographic approach, integrating this approach with inverse design optimization could enable even more intricate control within denser 3D spaces^14^. Looking ahead, this technology holds great promise for enhancing 3D imaging in mid-to-short-range LiDAR systems^15^ and could be paired with polarization cameras to capture high-dimensional visual data beyond the reach of conventional optical systems and sensors^16^.
